# Comparative Evaluation of Risk Assessment Models for Predicting Venous Thromboembolic Events in Cancer Patients with Implanted Central Venous Access Devices

**DOI:** 10.3390/cancers17203308

**Published:** 2025-10-14

**Authors:** Mohammad Ma’koseh, Heba Farfoura, Mahmoud Abunasser, Maryam El-Atrash, Anas Zayed, Renad Hamdan-Mansour, Zaid Abdel Rahman, Tala Ghatasheh, Mohammad Alshobaki, Mohammed J. Al-Jaghbeer, Hikmat Abdel-Razeq

**Affiliations:** 1 Department of Internal Medicine, King Hussein Cancer Center, Amman 11941, Jordanza.11040@khcc.jo (Z.A.R.);; 2 School of Medicine, University of Jordan, Amman 11942, Jordan; 3 Department of Radiology, King Hussein Cancer Center, Amman 11941, Jordan; 4 Office of Scientific Affairs and Research, King Hussein Cancer Center, Amman 11941, Jordan

**Keywords:** venous thromboembolic events, implanted central venous access devices, risk assessment models, survival

## Abstract

Background/Objectives: Cancer patients using implanted venous access devices (ICVADs) for chemotherapy are at increased risk of venous thromboembolism (VTE), but the performance of risk assessment models (RAMs) in this setting is understudied. This study evaluated VTE incidence, risk factors, and the predictive performance of the Khorana, COMPASS-CAT, and ONKOTEV models. Methods: We retrospectively reviewed records of adult cancer patients treated with chemotherapy via ICVADs. The cumulative incidence (CI) of VTEs was estimated using the Fine–Gray method, and RAM performance was assessed by sensitivity, specificity, predictive values, accuracy, and AUC. Overall survival (OS) was analyzed using Kaplan–Meier and log-rank tests. Results: A total of 446 patients were included. The most common cancers were colorectal (29.6%), gastric (26%), pancreatic (18.4%), and breast (13.9%). During a median follow-up of 16.5 months, VTEs occurred in 82 patients (18.4%), including 43 (9.6%) that were ICVAD-related. Median time to VTE was 117 days and 68 days for ICVAD-related events. The CI of VTEs was 9% at 1 year and 18.4% at 2 years. ONKOTEV showed the best performance (accuracy of 74.4%, specificity of 85.7%, and AUC of 0.607), with 1-year incidence higher in the high-risk group (28.5% vs. 12.4%, *p* < 0.001). In contrast, all RAMs showed limited ability for ICVAD-related VTEs. VTE was independently associated with inferior OS (HR 1.39, *p* = 0.037). Conclusions: Cancer patients with ICVADs face a substantial risk of early VTEs. Among evaluated RAMs, ONKOTEV performed best for overall but not ICVAD-related events. Prospective studies are needed to guide prophylaxis strategies using validated RAMs.

## Simple Summary

Patients with solid tumors receiving chemotherapy through implanted venous access devices are at increased risk of venous thromboembolism (VTE), but existing risk prediction models are not well validated for this group. We conducted a retrospective study of 446 patients with solid tumors to assess the incidence, timing, and predictors of VTE, and to evaluate the accuracy of the Khorana, COMPASS, and ONKOTEV risk assessment models. We observed an overall VTE rate of 18.4%, with nearly half of events being device-related and occurring early after insertion (median 68 days). Among the models assessed, the ONKOTEV score showed the best predictive performance, while all models showed limited value for device-related events. Importantly, VTE was independently associated with worse overall survival. These findings highlight the need for improved risk stratification and targeted, potentially time-limited prophylaxis strategies in cancer patients receiving chemotherapy through implanted devices.

## 1. Introduction

Venous thromboembolic events (VTEs) are a major complication in cancer patients, contributing to increased morbidity, mortality, and healthcare burden [1]. The prothrombotic state associated with malignancy is primarily driven by tumor-mediated coagulation activation, chemotherapy-induced endothelial injury, and immobility [2].

Central venous catheters (CVCs), commonly used for chemotherapy administration, further elevate the risk of VTEs due to mechanical vascular irritation and stasis [3,4]. Compared with external CVCs, implanted central venous access devices (ICVADs) offer several advantages, including being less visible and more acceptable for the patients, requiring less special care, and having a lower risk of complications, including VTEs, markedly improving patients’ quality of life [5].

However, the reported incidence of VTEs in cancer patients with ICVADs varies widely across the literature, depending on the study design, patient population, and methods used to detect thromboembolic events. Most studies focus specifically on catheter-related thrombosis, with incidence rates ranging from 0.3% to 11.5% [6,7]. Only a few studies have reported the overall incidence of VTEs, with estimates ranging from 11% to 15% [8,9].

Several risk assessment models (RAMs) have been developed to stratify cancer patients based on their risk for VTEs. The Khorana score [10], which assigns points according to tumor type, platelet and leukocyte counts, hemoglobin level, and body mass index, remains the most widely validated and utilized model in clinical practice [11] and is endorsed by several clinical practice guidelines [12,13]. However, it has shown limited sensitivity in some cancer subtypes and clinical contexts [14]. The Clinical Prediction Model to Assess the Risk of Venous Thromboembolism in Ambulatory Patients with Solid Tumors Receiving Chemotherapy (COMPASS-CAT) [15] expands risk estimation by including comorbidities, treatment exposures such as hormonal or anthracycline therapy, and recent hospitalization. The Oncology Thromboembolism (ONKOTEV) [16] model integrates clinical and anatomic factors—specifically metastatic disease, a prior VTE, a Khorana score >2, and vascular or lymphatic compression—to refine risk discrimination. Nevertheless, the utility of these models specifically in patients receiving chemotherapy through ICVADs remains inadequately studied.

Despite the well-recognized thrombotic risk in this setting, the role of prophylactic anticoagulation in patients with ICVADs remains questionable. Clinical trials evaluating routine thromboprophylaxis in cancer patients with CVCs have yielded mixed results, with no clear consensus on efficacy [17,18,19]. The heterogeneity of patient populations, variations in cancer types and stage, and inconsistent risk stratification methods may have contributed to these inconclusive findings.

Given these limitations, our study aims to evaluate the incidence and risk factors for VTEs in a cohort of cancer patients receiving chemotherapy via ICVADs. We also assessed the predictive performance of the Khorana, COMPASS, and ONCOTEV RAMs in this population, with the goal of identifying a model that can reliably guide prophylactic strategies and improve patient outcomes.

## 2. Materials and Methods

Adult patients (≥18 years) with pathologically confirmed diagnosis of solid cancer who had an ICVAD inserted, were treated, and were followed up at our institution between January 2021 and December 2023 were identified. Data were collected from the hospital databases and electronic medical records.

The following variables were recorded: age, sex, cancer type, personal history of VTE, myocardial infarction, stroke, diabetes, hyperlipidemia, peripheral vascular disease, smoking history, and treatments administered via the ICVAD. Baseline complete blood count and body mass index (BMI) at the time of ICVAD insertion were documented. Cancer stage was categorized as localized, locally advanced, or metastatic (as defined in the COMAPSS-CAT RAM or as a binary classification of localized/locally advanced vs. metastatic) and the presence of vascular or lymphatic compression was determined based on the most recent evaluation prior to device insertion. Patients receiving therapeutic or prophylactic anticoagulation at the time of ICVAD insertion were excluded. However, those receiving antiplatelet agents were not excluded.

The Khorana and ONKOTEV RAMs were calculated for each patient [8,13,14]. The Khorana score assigns points for cancer type, platelet count, hemoglobin level or use of erythropoiesis-stimulating agents, leukocyte count, and body mass index, classifying patients into low- (0 points), intermediate- (1–2 points), and high-risk (≥3 points) categories. The ONKOTEV score incorporates four factors: Khorana score >2, history of VTE, metastatic disease, and vascular or lymphatic compression, with risk groups defined as low (0 points), intermediate (1 point), and high risk (≥2 points). We first evaluated the performance of both scores according to their original three-category classification and then dichotomized them (low/intermediate vs. high) to better identify the subgroup of patients who may benefit from thromboprophylaxis. For the COMPASS-CAT score, all components were retained, including the 3 points assigned for the presence of a CVC, since all patients in our cohort had an ICVAD. Given this uniform presence of a heavily weighted risk factor, we explored the use of ROC curve analysis to identify an optimal cut-off point for risk stratification. However, the ROC curve did not yield a meaningful threshold with acceptable discrimination, as seen in Supplementary Figure S1. Therefore, patients were categorized into low-risk (score ≤9) and high-risk (score >9) groups. No routine imaging for screening of VTE was performed. VTE diagnosis was based on clinical suspicion and confirmed by radiological imaging—Doppler ultrasound for deep vein thrombosis (DVT) and computed tomography (CT) angiography for pulmonary embolism (PE) for symptomatic patients.

Patients were followed from the time of ICVAD insertion until death, last follow-up, or up to six months after device removal, whichever occurred later. VTE was defined as any event if diagnosed any time after ICVAD insertion and up to six months after its removal. VTEs were considered ICVAD-related if they occurred in anatomical proximity to the catheter or venous territory affected by the device. Major bleeding, as a complication of anticoagulation, was defined as fatal bleeding and/or symptomatic bleeding in a critical organ and/or bleeding causing a fall in hemoglobin level of ≥2.0 g/dL or leading to transfusion of two or more units of red blood cells.

Descriptive statistics were used to characterize patients at baseline. Continuous variables were presented as medians (range), and categorical variables were expressed as numbers (percentages). The cumulative incidence (CI) of VTE was estimated using the Fine and Gray method, accounting for competing risks. Death occurring before the diagnosis of VTE in patients who did not undergo ICVAD removal, or within six months of ICVAD removal without prior VTE, was considered a competing event. Group comparisons of CI were performed using Gray’s test, and independent predictors of VTE were identified using Fine–Gray sub distribution hazard regression. Overall survival was estimated using the Kaplan–Meier method and compared using the log-rank test. All *p*-values were two-sided, and values <0.05 were considered statistically significant. The predictive performance of the Khorana, COMPASS-CAT, and ONKOTEV scores was evaluated after dichotomization into low/intermediate- versus high-risk groups. Metrics included sensitivity, specificity, positive predictive value (PPV), negative predictive value (NPV), overall accuracy, balanced accuracy, and the area under the receiver operating characteristic curve (AUC). All metrics were reported with 95% confidence intervals.

## 3. Results

### 3.1. Patient Characteristics

A total of 446 patients were included in the analysis with a median age of 56 (range: 19–81) years and an almost equal sex distribution. The most common malignancies were colorectal (*n* = 132, 29.6%), gastric cancer (*n* = 116, 26%), pancreatic cancer (*n* = 82, 18.4%), and breast cancer (*n* = 62, 13.9%). A total of 29 (6.5%) patients received anthracycline-based or hormonal therapy, and 84 patients (18.8%) were treated with novel therapies, including immune checkpoint inhibitors and vascular endothelial growth factor receptor (VEGFR) inhibitors, as seen in Table 1.

At the time of ICVAD insertion, 136 (30.5%) patients had a localized disease, 63 (14.1%) had locally advanced disease, and 247 (55.4%) had metastatic disease. The median time from initial cancer diagnosis to ICVAD insertion was 1.2 (range: 0.5–177) months. Within 30 days prior to ICVAD insertion, 109 patients (24.4%) were hospitalized, and 71 (15.9%) had evidence of vascular or lymphatic compression on imaging. All ICVDs were silicon ports and were inserted into the subclavian vein.

### 3.2. Venous Thromboembolism

VTEs were reported in 82 (18.4%) patients, corresponding to an incidence of 0.46 events per 1000 catheter days (95% CI: 0.36–0.56). Among these, 43 (9.6%) were ICVAD-related (41 upper limb DVT and 2 both upper limb DVT and PE), corresponding to an incidence of 0.19 events per 1000 catheter days (95% CI: 0.13–0.25). Non-ICVAD related VTEs included isolated lower limb DVT in 17 (20.7%), lower limb DVT with PE in 2 patients (2.4%), isolated PE in 11 (13.4%) patients, and 9 patients (2%) had abdominal vein thrombosis: four in the inferior vena cava, three in the splenic veins, one in the hepatic vein, and one involving both the ovarian and renal veins. The distribution of thrombotic events varied according to primary tumor type. Among all VTE cases, the most frequent cancers were colorectal (34.2%), gastric (28.0%), and pancreatic (20.7%), followed by esophageal (6.1%), breast (7.3%), duodenal (2.4%), and anal cancer (1.2%). When restricted to ICVAD-related VTEs, most occurred in patients with colorectal (34.9%) and gastric cancers (32.6%), with additional cases observed in pancreatic (14.0%), esophageal (9.3%), and breast cancers (9.3%).

**Figure 1:**
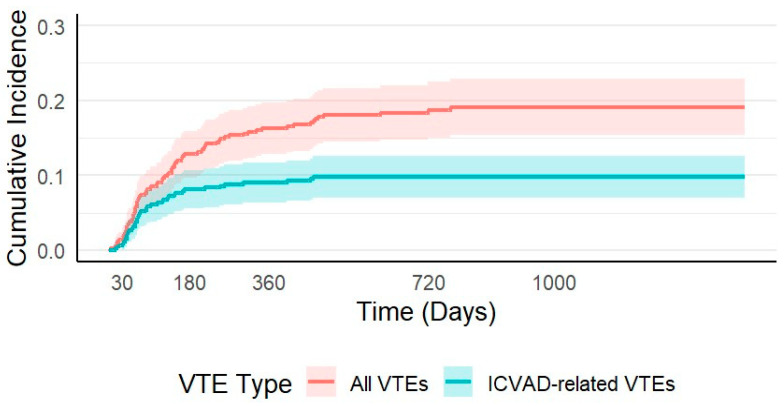
Cumulative incidence of all venous thromboembolic events (VTEs) (red line) and implanted central venous access device (ICVAD)-related VTEs (blue line) over time.

The median time from ICVAD insertion to the diagnosis of any VTE was 117 days (range: 3–768), while the median time to ICVAD-related VTE was shorter, at 68 days (range: 13–459). The CI of VTE progressively increased over time following ICVAD insertion. At 30 days, the CI of VTE was 0.9% (95% CI: 0.02–1.77%), rising to 5.84% (95% CI: 3.66–8.03%) at 90 days and 8.10% (95% CI: 5.56–10.65%) at 180 days. By 12 months (365 days), the CI reached 9.02% (95% CI: 6.35–11.69%) and continued to increase to 18.4% (95% CI: 14.4–22.2%) at two years. The cumulative incidence of ICVAD-related VTE followed a similar early rise. It was 0.9% (95% CI: 0.02–1.77%) at 30 days, 5.8% (95% CI: 3.66–8.03%) at 90 days, 8.1% (95% CI: 5.56–10.65%) at 180 days, and 9.0% (95% CI: 6.35–11.69%) at 360 days. However, by two years, the cumulative incidence of ICVAD-related VTE was 9.3% (95% CI: 6.55–12.01%), as seen in Figure 1.

In univariate analysis, the CI of VTE was significantly higher in patients with diabetes (1-year 20.7% vs. 14%; *p* = 0.02), metastatic disease (1-year 18.4% vs. 12.6%; *p* = 0.02), white blood cell count (WBC) > 11 × 10^9^/L (1-year 29.1% vs. 14.4%; *p* = 0.007), and vascular or lymphatic compression (1-year 29.9% vs. 13.1%; *p* = 0.01), Table 1.

In the multivariate analysis, vascular or lymphatic compression was the only factor that remained independently associated with an increased risk of VTE (HR 2.10, 95% CI 1.27–3.48; *p* = 0.0038). Other variables, including WBC > 11 × 10^9^/L, diabetes, and metastatic disease, did not reach statistical significance, Table 2.

Treatment of VTEs included low-molecular-weight heparin (LMWH; enoxaparin, *n* = 13; tinzaparin, *n* = 6; fondaparinux, *n* = 35) in 54 (65.9%) patients and direct oral anticoagulants (DOACs; apixaban, *n* = 22; rivaroxaban, *n* = 1) in 23 (28%) patients. Five patients (6.1%) did not receive anticoagulation because of bleeding risk or coagulopathy. The median duration of anticoagulation was 5.2 months (range: 1–12). Anticoagulation was complicated by bleeding in 10 patients (12.2%), of which 3 (3.6%) were major. One patient developed heparin-induced thrombocytopenia (HIT). Nine of the bleeding events occurred in patients treated with LMWH, and one occurred in a patient treated with a DOAC, with no statistically significant difference between groups (*p* = 0.271).

### 3.3. Risk Assessment Models

#### 3.3.1. All VTEs

**Figure 2:**
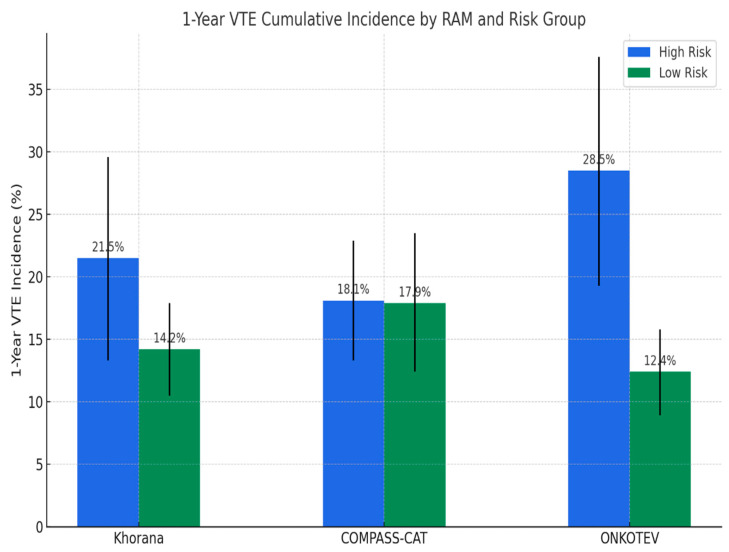
One-year cumulative incidence of VTE according to the Khorana (≥3 = high risk), COMPASS-CAT (>9 = high risk), and ONKOTEV (≥2 = high risk) models.

The performance of the Khorana, COMPASS-CAT, and ONKOTEV RAMs in predicting VTEs is summarized in Figure 2 and Table 3.

According to the Khorana score, 142 patients (31.8%) were classified as low risk, 205 (46%) as intermediate risk, and 99 (22.2%) as high risk, with 1-year cumulative incidences (CI) of VTEs of 12%, 16%, and 22%, respectively. There was no significant difference in CI between low- and intermediate-risk groups (*p* = 0.48), low- and high-risk groups (*p* = 0.97), or intermediate- and high-risk groups (*p* = 0.25). When dichotomized into low/intermediate versus high risk, the 1-year VTE incidence was 14.2% (95% CI, 10.5–17.9) compared with 21.5% (95% CI, 13.3–29.6) (*p* = 0.12). While specificity was relatively high (79.1%), sensitivity was limited (28.0%), and overall accuracy was 69.7%. The positive predictive value (PPV) was 23.2%, and the negative predictive value (NPV) was 83.0%, with a balanced accuracy of 53.6% and an AUC of 0.536. Only 28% of VTEs occurred in the high-risk category. Overall, the Khorana score demonstrated limited discriminatory performance, with modest accuracy and poor sensitivity in identifying high-risk patients.

The COMPASS-CAT score categorized 82 patients (18.4%) as high risk (score >9). The 1-year VTE incidence was 18.1% (95% CI, 13.3–22.9) in the high-risk group compared with 17.9% (95% CI, 12.4–23.5) in the low-risk group (*p* = 0.76). Although it showed higher sensitivity (57.3%) and NPV (81.9%), its specificity (43.4%), accuracy (45.9%), PPV (18.6%), and AUC (0.504) were the lowest among the three models, reflecting weak overall performance and minimal separation between risk groups.

The ONKOTEV score classified 145 patients (32.5%) as low risk, 197 (44.2%) as intermediate risk, and 104 (23.3%) as high risk, with 1-year cumulative incidences of 10.4%, 14.8%, and 28.5%, respectively. While there was no significant difference between low- and intermediate-risk groups (*p* = 0.12), the differences were significant between low- and high-risk groups (*p* < 0.001) and between intermediate- and high-risk groups (*p* = 0.004). When dichotomized into low/intermediate versus high risk, the 1-year VTE incidence was 12.4% (95% CI, 8.9–15.8) compared with 28.5% (95% CI, 19.3–37.6) (*p* < 0.001). The ONKOTEV score demonstrated the best overall performance, with the highest accuracy (74.4%), specificity (82.4%), PPV (33.3%), and AUC (0.607), along with a sensitivity of 39% and NPV of 85.7%. Overall, 39% of VTEs occurred in the high-risk group, showing the best discriminatory performance among the three models with clearer separation between low- and high-risk patients.

#### 3.3.2. ICVAD Related VTEs

For ICVAD-related VTEs, the Khorana score classified patients into low-, intermediate-, and high-risk groups with 12-month cumulative incidences of 4.9% (95% CI, 1.4–8.5), 11.3% (95% CI, 6.9–15.6), and 10.2% (95% CI, 4.2–16.3), respectively. Differences between groups were not statistically significant (intermediate vs. low: sHR 1.91, 95% CI 0.89–4.11, *p* = 0.097; high vs. low: sHR 1.64, 95% CI 0.67–4.02, *p* = 0.280). When dichotomized into low/intermediate versus high risk, the 12-month incidence was 8.7% (95% CI, 5.7–11.6) compared with 10.2% (95% CI, 4.2–16.3) (sHR 1.07, 95% CI 0.53–2.16, *p* = 0.85).

Similarly, the ONKOTEV score yielded 12-month cumulative incidences of 7.6% (95% CI, 3.3–11.9), 9.2% (95% CI, 5.1–13.3), and 10.7% (95% CI, 4.7–16.7) in the low-, intermediate-, and high-risk groups, with no significant differences (intermediate vs. low: sHR 1.27, 95% CI 0.60–2.67, *p* = 0.53; high vs. low: sHR 1.72, 95% CI 0.77–3.87, *p* = 0.19). Dichotomization into low/intermediate versus high risk showed a 12-month incidence of 8.3% (95% CI, 5.4–11.2) compared with 11.6% (95% CI, 5.1–18.0) (sHR 1.67, 95% CI 0.87–3.20, *p* = 0.13).

For the COMPASS-CAT score, the 12-month incidence was 9.9% (95% CI, 5.6–14.1) in the low-risk group and 8.4% (95% CI, 4.9–11.8) in the high-risk group, with no significant difference (sHR 0.89, 95% CI, 0.49–1.62, *p* = 0.70).

In terms of predictive performance, the ONKOTEV score achieved the highest accuracy (74.7%) and specificity (79.4%), with modest sensitivity (30.2%) and an AUC of 0.548. The Khorana score showed similar accuracy (72.6%) but lower sensitivity (23.3%) (AUC 0.506), while the COMPASS-CAT score had the highest sensitivity (53.5%) but the lowest specificity (42.9%) and accuracy (43.9%), with an AUC of 0.482. Overall, none of the RAMs showed meaningful discriminatory ability for predicting ICVAD-related VTEs.

### 3.4. Survival Outcomes

**Figure 3:**
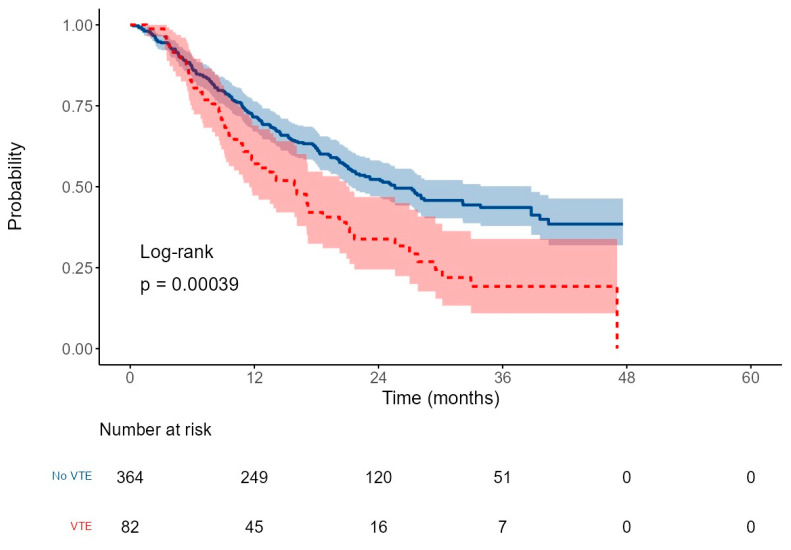
Overall survival in patients with (red line) or without (blue line) VTE, with 95% confidence intervals (CI).

After a median follow-up of 16.5 months (range: 0.2–47.6), the median overall survival (OS) for the entire cohort was 22.2 months (95% CI: 20.2–27.5), with a 2-year OS rate of 48.8%. Patients who developed VTEs had significantly shorter survival, with a median OS of 16.1 months (95% CI: 11.5–21.2), compared to 25.6 months (95% CI: 21.3–38.8) in those without VTE. The corresponding 2-year OS rates were 33.8% versus 52.3%, respectively (HR: 1.7, *p* < 0.001), Figure 3.

In the univariate analysis, several additional variables showed significant associations with OS, including history of DM (2-year OS 39% vs. 52.3%, *p* = 0.02), low body mass index (BMI) (*p* < 0.0001), metastatic disease (2-year OS 28.6% vs. 74.2%, *p* < 0.001), vascular compression (2-year OS 26.4% vs. 52.9%, *p* < 0.001), and use of novel therapy (2-year OS 65.3% vs. 45.3%, *p* = 0.049), as seen in Supplementary Table S1.

In the multivariate analysis, metastatic disease (HR 4.6, *p* < 0.001), use of novel therapy (HR 0.47, *p* < 0.001), BMI (HR 0.94, *p* < 0.001), and VTEs (HR 1.39, *p* = 0.037) remained statistically significant, as seen in Supplementary Table S2.

## 4. Discussion

In this retrospective study, cancer patients receiving chemotherapy via ICVADs had a high incidence of VTEs (18.4%), with nearly half (9.6%) being device related. Among the three evaluated RAMs, only the ONKOTEV score demonstrated significant discriminatory ability to identify patients at higher risk. Additionally, the occurrence of VTE was independently associated with worse OS.

Limited number of studies have specifically reported the overall incidence of VTEs in cancer patients with ICVADs. In the ONCOCIP trial, the overall VTE rate was 13.1% [8], and Hohl Moinat et al. reported a one-year incidence of 15.3% [9]. The relatively higher rate observed in our cohort (18.4%) likely reflects the predominance of gastrointestinal malignancies, particularly gastric and pancreatic cancers, which are known to be associated with elevated thrombotic risk [8]. The 9.6% incidence of ICVAD-related thrombosis also falls at the higher end of reported ranges (0.3–11.5%) [20,21,22,23], indicating the clinically significant burden of these devices on the development of VTEs in our cohort.

The Khorana RAM showed limited ability to identify high-risk patients. Although VTE incidence increased with higher scores (from 14.2% in low-risk to 21.5% in high-risk), the difference was not statistically significant (*p* = 0.12), and only 28% of VTEs occurred in the high-risk group. These findings align with a meta-analysis by Mulder et al., which reported that only 23.4% of VTEs occurred in patients classified as high risk [24]. The score’s limited utility in our population may be explained by its underperformance in gastrointestinal cancers [24,25] and that it does not include catheter-related factors.

The COMPASS-CAT score demonstrated a relatively higher sensitivity (57.3%) and negative predictive value (81.9%), but it had lower specificity (43.4%) and the lowest overall accuracy among the three models (46.0%). Although the COMPASS-CAT model classified a similar proportion of patients as high risk (18.2%) compared to Khorana (22.2%) and ONKOTEV (21.5%), its performance remained inferior. This indicates that the model’s limited predictive utility in our cohort is not related to the size of the high-risk group but rather to inadequate risk discrimination and limited relevance of certain score components. The score was originally developed and validated in a population primarily composed of patients with breast, colorectal, and lung cancers [26,27], while nearly half of our cohort had gastric or pancreatic malignancies. Moreover, components that are heavily weighted in the score, such as anthracycline and hormonal therapies, were rarely used in our population, further diminishing its applicability.

By contrast, the ONKOTEV score demonstrated the best overall performance. It achieved the highest accuracy (74.4%) and specificity (85.7%) among the three models, with moderate sensitivity (33.3%) and a PPV of 39%. These results are consistent with previous validation studies in patients with different types of solid tumors [28], in pancreatic cancer [29], and in a recent study where ONKOTEV outperformed the Khorana score in guiding individualized prevention of VTEs in outpatients with cancer [30]. To our knowledge, this is the first study to evaluate the ONKOTEV model specifically in patients with ICVADs. Its improved performance likely reflects the inclusion of metastatic disease and vascular or lymphatic compression; two variables significantly associated with VTE in our analysis. However, the model missed a substantial proportion of VTEs, suggesting that further refinement is warranted.

When evaluating ICVAD-related VTEs separately, none of the three RAMs, including ONKOTEV, demonstrated meaningful discriminatory ability. This limitation likely reflects the fact that none of these models incorporate catheter-related variables, which are well-established determinants of thrombosis risk. Previous studies have shown that factors such as insertion site, number of puncture attempts, catheter tip position, lumen size, and operator experience significantly influence the risk of catheter-associated thrombosis [5,21]. Our findings are therefore consistent with prior reports that conventional RAMs are inadequate for predicting catheter-related events [3,6]. To address this gap, future models should integrate device- and procedure-specific factors alongside patient- and cancer-related variables, as also emphasized by international guidelines [13]. Such refinements may allow more accurate identification of patients at highest risk and support individualized strategies for thromboprophylaxis in those with ICVADs.

In our analysis, several factors such as diabetes, metastatic disease, leukocytosis, and vascular or lymphatic compression were associated with higher VTE risk in the univariate analysis. However, only vascular or lymphatic compression remained independently significant in the multivariate model. This highlights the importance of local anatomic factors in driving thrombosis risk, while systemic or clinical features may act as surrogate markers of tumor burden. These findings are consistent with prior studies linking advanced disease and venous stasis with cancer-associated thrombosis [8,24,25].

An important observation in our study was the timing of VTEs. The cumulative incidence of ICVAD-related VTE plateaued by 6 months (8.1%), whereas overall VTE risk continued to rise, reaching 18.4% by two years. This pattern suggests that catheter-associated thrombosis is predominantly an early complication, while longer-term risk is driven by disease burden and ongoing therapy. These findings support the six-month time-limited approach to prophylaxis implemented in prospective trials like CASSINI [31] and AVERT [32], emphasizing the importance of ongoing individualized risk assessment throughout cancer care.

Although the development of VTEs has been linked to more aggressive tumor biology, advanced stage, and certain cancer therapies [33], multiple studies have reported an independent association between VTEs and increased mortality. For example, the prospective Scandinavian Thrombosis and Cancer (STAC) study demonstrated a 3.4-fold increase in the risk of death among cancer patients who developed VTEs, regardless of cancer type or severity [34]. Our findings are consistent with these observations.

However, in the era of novel therapies, the impact of VTEs on survival may vary depending on tumor type, disease stage, and the timing of VTEs’ occurrence. A recent meta-analysis, for instance, found no significant association between cancer-related VTEs and OS in breast cancer patients [35], while in pancreatic cancer, only early VTEs (within three months of diagnosis) were associated with worse survival outcomes [36]. Individualized risk assessment and additional studies are warranted to clarify the prognostic role of VTEs across different cancer types and treatment settings.

Among patients who developed VTEs, 6.1% did not receive anticoagulation, while 12.2% experienced bleeding complications during treatment, including major bleeding in 3.6%. These rates are comparable to those reported in real-world studies [13]. Previous studies have reported a non-significant increase in major bleeding with DOACs compared to LMWH and higher rates of non-major bleeding with DOACs [24]. Anthracyclines may also contribute indirectly to bleeding through chemotherapy-induced thrombocytopenia or mucosal injury [37]. In our cohort, bleeding occurred more frequently in patients treated with LMWH compared to DOACs, although this difference was not statistically significant, likely due to the small number of events. Only three patients who developed VTE received anthracycline-based therapy, and none of these three experienced bleeding, which limited further analysis. These findings highlight the importance of balancing thrombotic and hemorrhagic risks, particularly in patients with gastrointestinal cancers.

Although this study provides one of the few real-world evaluations comparing three RAMs in cancer patients with ICVADs, it has important limitations that include its retrospective nature, potential underreporting of asymptomatic VTEs, and the single-center design, which may limit generalizability. The cancer distribution in our cohort, which reflects local ICVAD use patterns rather than the general cancer population, may also have influenced the performance of risk models such as the Khorana and ONCOTEV scores. In addition, biomarker-based scores such as CATMiCAS (Cancer Associated Thrombosis, risk prediction model integrating Clinical and biological factors such as D-dimer) could not be assessed because D-dimer testing was not routinely performed at diagnosis or ICVAD insertion in our cohort. Moreover, nursing management of the catheter—such as adherence to flushing protocols, dressing changes, and infection prevention practices—was not captured in this analysis. These factors may influence thrombotic risk and represent another area for future investigation.

## 5. Conclusions

Cancer patients receiving chemotherapy through ICVADs are at increased risk of VTEs, which was independently associated with worse overall survival. Among the evaluated risk models, the ONKOTEV score demonstrated the highest predictive accuracy in this population, although its sensitivity remained limited. Further studies are needed to assess its role in guiding prophylactic anticoagulation strategies and improving outcomes in high-risk patients.

## Figures and Tables

**Table 1:** Patient’s characteristics and univariate analysis for cumulative incidence (CI) of VTEs.

Variable	Number (%)	12-Month VTE CI (95% CI)	HR (95% CI)	*p*-Value
Age ≥ 60 years	124 (34%)	18.8% (12.6–25.0%)	1.24 (0.80–1.93)	0.33
Sex				
Female	235(52.7%)	15.3% (10.4–20.2%)	1.00 (0.65–1.54)	0.995
Male	211 (47.3%)	16.3% (11.5–21.0%)		
Current smoker	138 (31%)	18.9% (12.3–25.4%)	1.34 (0.83–2.17)	0.23
BMI ≥ 30 kg/m^2^	118 (26.5%)	18.3% (11.0–26.1%)	0.85 (0.43–1.70)	0.65
Antiplatelet	49 (11%)	18.7% (7.5–29.8%)	1.13 (0.59–2.14)	0.7
Time from cancer diagnosis to ICVAD insertion <6 months	253 (79.1%)	16.6% (12.7–20.5%)	1.07 (0.59–2.14)	0.33
Comorbidities				
Diabetes	122 (27.4%)	20.7% (13.4–27.9%)	1.70 (1.09–2.64)	0.02
Hypertension	153 (34.4%)	17.7% (11.6–23.8%)	1.19 (0.77–1.87)	0.43
Peripheral vascular disease	6 (1.3%)	0% (0–0%)	1.43 (0.43–4.78)	0.24
Stroke	4 (0.9%)	0% (0–0%)	1.27 (0.21–7.67)	0.82
Coronary artery disease	33 (7.4%)	18.2% (4.8–31.6%)	1.17 (0.55–2.50)	0.68
Hyperlipidemia	56 (12.6%)	12.5 (3.8–21.2%)	0.85 (0.43–1.70)	0.69
≥2 comorbidities	49 (11%)	16.4% (10.3–22.6%)	1.01 (0.93–1.11)	0.76
Cancer site				
Gastric	116 (26%)	19.3% (12.0–26.6%)	1.14 (0.71–1.84)	0.58
Pancreatic	82 (18.4%)	18.4% (9.9–26.9%)	1.22 (0.71–2.10)	0.44
Colorectal	132 (29.6%)	16.7% (10.3–23.1%)	1.32 (0.84–2.07)	0.23
Breast	62 (13.9%)	8.1% (1.2–14.9%)	0.45 (0.20–1.04)	0.063
Head and neck	19 (4.3%)	0% (0–0%)	0 (0–0)	<0.001
Esophageal	22 (4.9%)	15.9% (12.4–19.4%)	1.28 (0.51–3.21)	0.6
Others *	13 (2.9%)	0% (0–0%)	0.74 (0.21–2.68)	0.65
High risk sites according to Khorana RAM	198 (44%)	19.4% (13.9–25.0%)	1.26 (0.82–1.94)	0.28
Localized disease	199(44.6%)	12.6% (8.0–17.3%)	1.71 (1.08–2.72)	0.023
Metastatic disease	247 (55.4%)	18.4% (13.5–23.2%)		
Localized	136(30.5%)	13.3% (7.5–19.0%)	1.18 (0.92–1.52)	0.2
Locally advanced or metastatic	310 (69.5%)	16.9% (12.7–21.1%)		
Vascular or lymphatic compression	71 (15.9%)	29.9% (19.1–40.8%)	2.44 (1.47–4.06)	<0.001
No vascular or lymphatic compression	375 (84.1%)	13.1% (9.7–16.6%)		
WBC > 11 × 10^9^/L	42 (9.4%)	29.1% (15.0–43.2%)	2.19 (1.24–3.86)	0.007
Hb < 10 g/dL	69 (15.5%)	17.6% (8.5–26.8%)	1.27 (0.73–2.21)	0.4
Platelets ≥ 350 × 10^3^/µL	111 (24.9%)	15.3% (8.6–22.1%)	0.98 (0.60–1.61)	0.93
Hospitalization	109 (24.4%)	13.0% (6.6–19.4%)	0.82 (0.48–1.40)	0.46
Anthracycline or hormonal therapy	29 (6.5%)	6.9% (0–16.3%)	0.89 (0.74–1.08)	0.23
Novel therapy	84 (18.8%)	14.5% (6.8–22.1%)	0.97 (0.55–1.69)	0.9
Line infection	27 (8.3%)	13.5% (2.3–24.7%)	1.31 (0.68–2.52)	0.42

* Other sites include the following: Duodenum (4), Gynecological (2), Appendix (2), Lung (1), Cholangiocarcinoma (1), Kidney (1), and Sarcoma (2). Abbreviations: BMI: body mass index; ICVAD: implanted central venous access device; RAM: risk assessment model; WBC: white blood cell count; Hb: hemoglobin.

**Table 2:** Multivariate analysis of risk factors for venous thromboembolism in cancer patients with ICVADs.

Variable	HR (95% CI)	*p*-Value
WBC > 11 × 10^9^/L	1.72 (0.95–3.11)	0.073
Diabetes	1.44 (0.92–2.27)	0.110
Metastatic disease	1.37 (0.85–2.20)	0.190
Vascular compression	2.10 (1.27–3.48)	0.0038

Abbreviations: WBC, white blood cell count; HR, hazard ratio; CI, confidence interval; ICVAD, implanted central venous access device.

**Table 3:** Performance metrics of risk assessment models in all VTEs and in ICVAD-related VTEs.

Metric	Khorana % (95% CI)	ONCOTEV % (95% CI)	COMPASS-CAT % (95% CI)
**All VTEs**			
Sensitivity	28.0 (19.5–38.6)	39.0 (29.2–49.8)	57.3 (46.5–67.5)
Specificity	79.1 (74.7–83.0)	82.4 (78.2–86.0)	43.4 (38.4–48.5)
PPV	23.2 (16.0–32.5)	33.3 (24.7–43.2)	18.6 (14.3–23.8)
NPV	83.0 (78.7–86.6)	85.7 (81.7–89.0)	81.9 (75.8–86.7)
Accuracy	69.7 (65.3–73.8)	74.4 (70.2–78.3)	46.0 (41.4–50.6)
AUC	0.536	0.607	0.504
**ICVAD-related VTEs**			
Sensitivity	23.3 (12.0–39.0)	30.2 (17.0–46.0)	53.5 (38.0–69.0)
Specificity	77.9 (74.0–82.0)	79.4 (75.0–83.0)	42.9 (38.0–48.0)
PPV	10.1 (5.0–18.0)	13.5 (7.0–22.0)	9.1 (6.0–13.0)
NPV	90.5 (87.0–93.0)	91.4 (88.0–94.0)	89.6 (84.0–94.0)
Accuracy	72.6 (68.0–77.0)	74.7 (70.0–79.0)	43.9 (39.0–49.0)
AUC	0.506	0.548	0.482

Abbreviations: RAM = risk assessment model; VTE = venous thromboembolism; ICVAD = implanted central venous access device; PPV = positive predictive value; NPV = negative predictive value; AUC = area under the receiver operating characteristic curve.

## Data Availability

The data presented in this study are available on request from the corresponding author.

## Supplementary Materials

The following supporting information can be downloaded at: https://www.mdpi.com/article/10.3390/cancers17203308/s1, Figure S1: Receiver operating characteristic (ROC) curve for the COMPASS-CAT score in predicting venous thromboembolism in cancer patients with ICVADs. Table S1: Univariate analysis for overall survival. Table S2: Multivariate analysis for overall survival.

## Abbreviations

The following abbreviations are used in this manuscript:

AUC	Area under the receiver operating characteristic curve
BMI	Body mass index
CI	Cumulative incidence
CVC	Central venous catheter
DOAC	Direct oral anticoagulant
DVT	Deep vein thrombosis
Hb	Hemoglobin
HR	Hazard ratio
ICVAD	Implanted central venous access device
LMWH	Low-molecular-weight heparin
NPV	Negative predictive value
OS	Overall survival
PE	Pulmonary embolism
PPV	Positive predictive value
RAM	Risk assessment model
ROC	Receiver operating characteristic
VTE	Venous thromboembolic event(s)
WBC	White blood cell count
