# Supplementary material for: Comparative Evaluation of Risk Assessment Models for Predicting Venous Thromboembolic Events in Cancer Patients with Implanted Central Venous Access Devices
Source: Cancers (Basel). 2025 Oct 14;17(20):3308. doi: 10.3390/cancers17203308 (PMC12564628; doi:10.3390/cancers17203308)
Supplement: Supplementary file 1 [file cancers-17-03308-s001.zip › cancers-3871340-supplementary.pdf]

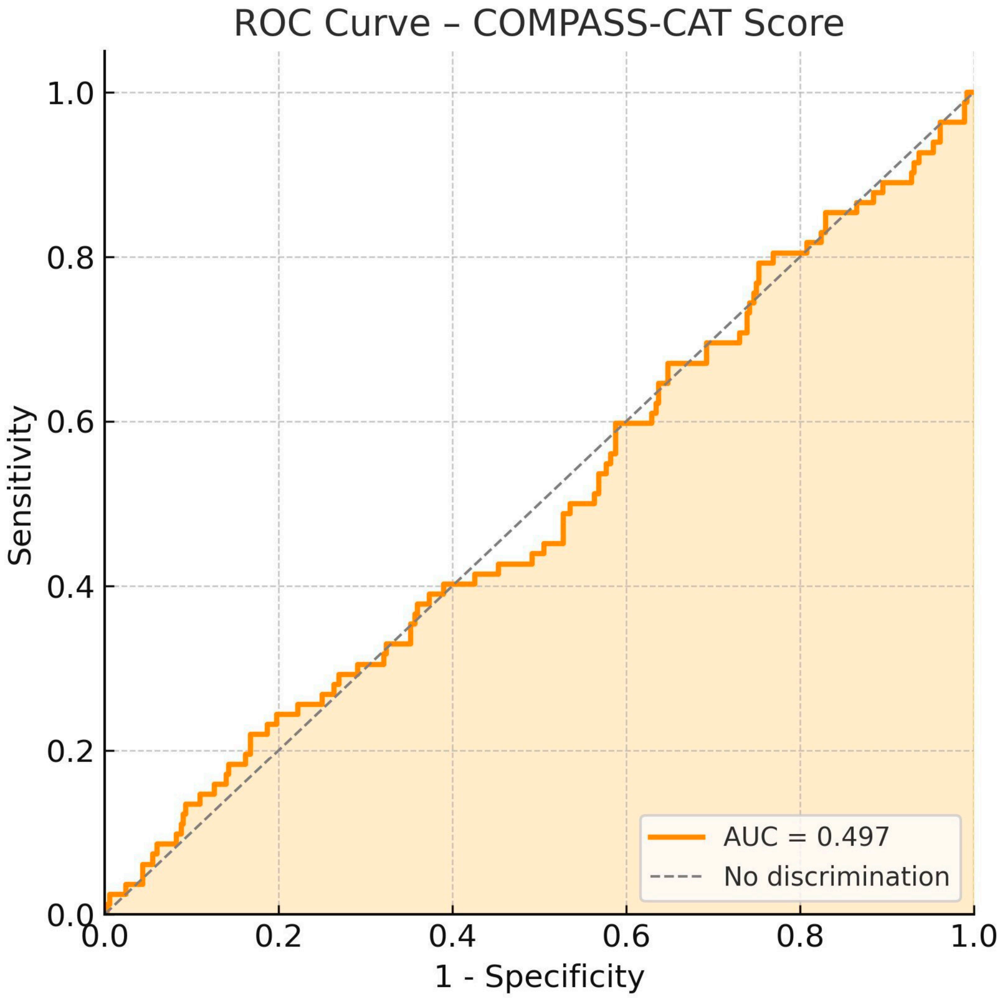

Figure S1: Receiver operating characteristic (ROC) curve for the COMPASS-CAT score in predicting venous thromboembolism in cancer patients with implanted venous access devices (ICVADs).

Supplementary Table S1: Univariate analysis for overall survival (OS)

Variable	2-year OS%	HR (95% CI)	P value
Age <60<br>Age ≥60	51.3%<br>44.0%	1.23 (0.94–1.60)	0.134
Female<br>Male	52.2%<br>45.0%	1.24 (0.96–1.60)	0.100
Non-smoker<br>Smoker	48.8%<br>48.7%	1.05 (0.79–1.38)	0.741
Body mass index		0.94 (0.91–0.96)	<0.001
No diabetes<br>Diabetes	52.3%<br>39.0%	1.38 (1.05–1.82)	0.022
No coronary artery disease<br>Coronary artery disease	48.7%<br>50.9%	1.12 (0.68–1.84)	0.654
No peripheral vascular disease<br>peripheral vascular disease	48.6%<br>60.0%	0.52 (0.13–2.11)	0.364
No Hypertension<br>Hypertension	47.9%<br>50.4%	0.97 (0.74–1.28)	0.852
No Hyperlipidemia<br>Hyperlipidemia	48.9%<br>46.8%	0.88 (0.58–1.32)	0.528
Non-metastatic<br>Metastatic	74.2%<br>28.6%	4.25 (3.13–5.78)	<0.001
No vascular or lymphatic compression<br>Vascular or lymphatic compression	52.9%<br>26.4%	2.23 (1.63–3.04)	<0.001
No novel therapy<br>Novel therapy	45.3%<br>63.3%	0.71 (0.50–1.00)	0.049
No VTE<br>VTE	52.3%<br>33.8%	1.70 (1.26–2.29)	<0.001

Abbreviation: VTE: Venous thromboembolic event

Supplementary Table S2: Multivariate analysis for overall survival (OS).

Variable	HR (95% CI)	P value
Diabetes	1.30 (0.98–1.72)	0.068
Metastatic disease	4.62 (3.35–6.38)	<0.001
Vascular or lymphatic compression	1.26 (0.91–1.75)	0.160
Novel therapy	0.47 (0.33–0.68)	<0.001
VTE	1.39 (1.02–1.88)	0.037
Body mass index	0.94 (0.92–0.97)	<0.001

Abbreviation: VTE: Venous thromboembolic event
